# Scandium Recovery from Aqueous Solution by Adsorption Processes in Low-Temperature-Activated Alumina Products

**DOI:** 10.3390/ijms231710142

**Published:** 2022-09-04

**Authors:** Diana Daminescu, Narcis Duţeanu, Mihaela Ciopec, Adina Negrea, Petru Negrea, Nicoleta Sorina Nemeş, Adina Berbecea, Gheorghe Dobra, Sorin Iliev, Lucian Cotet, Alina Boiangiu, Laurentiu Filipescu

**Affiliations:** 1 Faculty of Industrial Chemistry and Environmental Engineering, Polytechnica University of Timişoara, Victoriei Square, no. 2, 300006 Timisoara, Romania; 2 Renewable Energy Research Institute-ICER, Polytechnica University of Timişoara, Gavril Musicescu Street, no. 138, 300774 Timisoara, Romania; 3 Soil Sciences Department, Banat’s University of Agricultural Sciences and Veterinary Medicine “King Mihai I of Romania” from Timisoara, Calea Aradului, no. 119, 300645 Timisoara, Romania; 4 Alro, S.A., Pitesti Street, no.116., 230048 Slatina, Romania; 5 Alum, S.A., Isaccei Street no. 83, 820228 Tulcea, Romania; 6 Faculty of Chemical Engineering and Biotechnology, University Politehnica Bucharest, Polizu Street, no. 1-7, 011061 Bucharest, Romania

**Keywords:** scandium, activated alumina, adsorption, kinetic and thermodynamic studies

## Abstract

In this paper, we studied the scandium adsorption from aqueous solutions on the surface of low-temperature-activated alumina products (GDAH). The GDAH samples are industrially manufactured, coming from the Bayer production cycle of the Sierra Leone bauxite as aluminium hydroxide, and further, by drying, milling, classifying and thermally treating up to dehydroxilated alumina products at low temperature. All experiments related to hydroxide aluminium activation were conducted at temperature values of 260, 300 and 400 °C on samples having the following particle sizes: <10 µm, 20 µm, <45 µm and <150 µm, respectively. The low-temperature-activated alumina products were characterised, and the results were published in our previous papers. In this paper, we studied the scandium adsorption process on the above materials and related thermodynamic and kinetic studies.

## 1. Introduction

The continuous growth of industries based on limiting the consumption of materials and energy has imposed accelerated demands for specific raw materials, especially for the production of new rare metal elements. Most economically advanced countries consider rare metals resources and their production technologies as “critical”, based on their supply risks and economic importance. So far, rare metals’ unique physical and chemical properties have made them indispensable for growing new technologies, critical for the evolution of the new digital and green industry. Thus, rare metals’ importance in the production of electronics, regenerable energy equipment, lightweight electric vehicles and other applications has produced an increased demand for new raw materials and secondary resources obtained by the low cost of rare metal recovery. Some systematic reviews on this subject may be found in the recent literature [1]. However, in addition to the benefits of industrial use of lanthanides, there are many negative environmental impacts associated with both the mining of raw material and rare metals production, as well as with rare metal conversion to finished products. Moreover, these problems are related to a huge accumulation of wastes, debris, residues and garbage, but also by increasing other environmental hazards. Studies and technical reports indicate that the chemicals used in the refining process of rare metals have been responsible for the occupational diseases and contamination of local residential areas, water pollution and agricultural land destruction. Occupational safety and public health risks related to rare metals must be addressed and analysed for every metal in all the process stages: mining, transportation, processing and disposal, storage of waste and decommissioning of the equipment and production units. Many research papers targeting these issues recommend recovery instead of opening new mining operation units and the rapid return, as much as possible, toward a circular economy [1,2,3,4,5,6,7,8,9,10,11,12,13,14,15,16,17,18,19,20,21,22,23,24,25,26,27,28,29,30,31,32,33,34,35,36,37,38,39,40]. Scandium is one of the most valuable metals in the rare metals category. Therefore, all the above recommendations refer especially to this metal. Besides the specific properties and applications similar to other rare metal groups, scandium has some particularly significant properties, such as low density, high hardness and tensile strengths and low elasticity modulus, which makes scandium a good alloy with aluminium to produce the alloys aluminium-scandium, used in the production of minor components. In this type of alloy, the content of 0.1% and 0.5% of scandium improves mechanical strength, corrosion resistance and weldability. Other application developments of scandium are: (a) stabiliser of electrolyte (zirconia) in SOFCs (solid oxide fuel cells) in combination with yttrium, having an effect of lowering the working temperature, as well as increasing the operating time of the cell and its power; (b) adjuvant for increasing the mechanical strength of titanium-scandium ceramics, used to enlarge the weldability and the mechanical strength of Al-Sc alloys; (c) basic components in the preparation of the laser material Gd3Sc2Ga3O12 (gadolinium scandium gallium garnet, GSGG), which is a more efficient material than Nd-doped yttrium aluminium garnet laser in ferrites and garnets containing scandium, and is used primarily in magnetically controlled switches in computers by modulating the light passing through the garnet, as well as though microwave equipment; (d) main component in mercury vapour high-intensity lights to create natural light, due to its broad emission spectrum that generates a “daylight” effect, suitable for camera lighting, movie and television studio lights; (e) ionic activator in TV or computer monitors as part of the typical host materials Sc2O3 and ScVO4, while ZnCdS2, activated with a mixture of silver and scandium, are creating a red phosphorescent colour fitted for use in television displays [41]. Due to scandium scarcity and its limited production, this metal is from far away and is one of the most expensive rare metals. In the last years, prices for 99.99% pure scandium fluctuated between USD 15,000 and USD 20,000 per kilogram. However, because of the global availability of limited amounts of this material and also due to the limited market for scandium, an impact of increasing prices might be possible at any given time.

The technical literature provides an overview of various possibilities for intensifying the scandium recovery process to achieve higher scandium production than conventional mineral processing technology. In recent years, published papers contain valuable ideas for new ways of approaching the intensive development of scandium recovery processes. Common topics such as absorption, precipitation and crystallisation of scandium compounds [42,43,44], liquid–liquid extraction with organic solvents [45,46], over-refined recovering technologies [23,47,48], biotechnologies [49] and advanced technologies (nanotube, carbon materials, composite materials and ionic liquids) [50,51,52,53,54,55,56,57] are predicting accelerated progress in the early future of scandium recovery processes from secondary sources.

The present paper studies scandium adsorption from pure scandium nitrate solutions in low-temperature-activated alumina products, whose properties have been largely presented in our previous papers [54,58,59]. The precursor of the low-temperature-activated alumina product is the aluminium hydroxide dried, milled and classified, and manufactured at Vimetco Alum SA Tulcea, Romania, after the implementation of the project “Endow the Research and Development Department of SC ALUM SA Tulcea with independent, efficient research facilities to support the economic competitiveness and business development”, a project co-funded by the European Regional Development Fund through the Competitiveness Operational Program 2014–2020. The samples of low-temperature-activated alumina products used in this paper are from the same batch as the ones used in our last paper [60]. The goals are similar in both studies, to determine the maximum adsorption capacity of low-temperature-activated alumina products, to identify the best adsorbent performances and to evaluate correlations between adsorbent properties and adsorbent performances. The only difference is that, in our previous paper, the adsorbate was silver, and in this paper, the adsorbate was scandium. Moreover, it will be interesting to compare the kinetics data for the adsorption of two ions with distinct mass differences on the same adsorbents.

## 2. Results and Discussions

### 2.1. Low-Temperature-Activated Alumina Products Characterisation

The most important characteristic properties of low-temperature-activated alumina products are BET specific surface, Langmuir specific surface, average pore width, mineralogical phases: Gibbsite, Bayerite, Boehmite, Gamma-Al2O3 and their crystallinity (or amorphous phase content) and the particle size distribution (naming each product). Previous studies [54,58] show that all of these characteristics are controlled by two parameters (calcination temperature and particle size), which act throughout the thermal program of each group of samples. All these characteristic properties are given in Table 1 and Table 2.

Sample reactivity is expressed in Table 1 and Table 2 by specific surface, average pore width and crystalline/amorphous phase ratios. From these tables, it is easily observable that sample activation started at a significant rate at 300 °C and 400 °C when only 1–10% gibbsite remained unconverted into other crystalline or amorphous phases. Therefore, the thermal program changes the crystalline/amorphous ratio in the samples and the changes in the crystalline/amorphous ratio cause, in turn, deep modifications in the specific properties of all low-temperature-activated alumina products, presented in Table 1 and Table 2, including some more efficient scandium adsorption processes. Therefore, the scandium adsorption process and its kinetics should always be related to the parameters from Table 1 and Table 2. In this paper, the representative samples of low-temperature-activated alumina products (GDAH-03-300, GDAH-03-400, GDAH-04-300 and GDAH-04-400, according to their specific properties) were selected for a trial concerning the scandium adsorption from aqueous solutions.

### 2.2. Zero Charge Point, pH_pZc_

**Figure 1:**
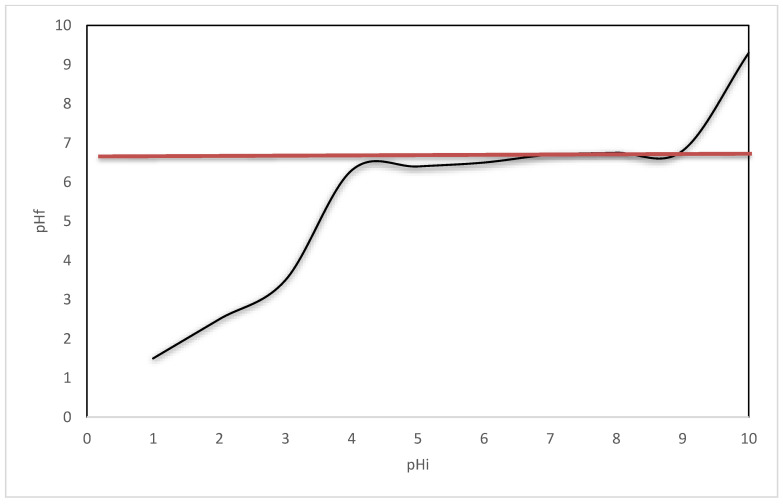
pHpzc experimental determination.

The zero charge point (pZc) defines a solution in which the surface density of positive charges (contribution of cations) equals that of negative charges (contribution of anions), particularly in terms of pH values [61,62,63]. Actually, this indicates the trend of the positive or negative charge surface depending on the pH. The pZc was determined using a potentiometric method [64] and a graphical representation of pH_final_ vs. pH_initial_. For determination of this parameter, an amount of 0.1 g of adsorbent material, GDAH-3-300, was mixed with 25 mL of 0.1 N KCl solution at 200 rpm and 298 K temperature, using a water bath with thermosetting and stirring, Julabo SW23 type. The pH of the KCl solutions was adjusted in the range of 2–12, using NaOH solutions with the concentrations 0.05 N and 2 N (obtained from pellets Merck Sigma Aldrich) and HNO_3_ 63% solutions (Carl Roth, Karlsruhe, Germany) diluted up to concentrations between 0.05 N and 2 N. The samples were filtered, and the pH of the resulting solution was then determined using a pH meter (METTLER TOLEDO, SevenCompact, S 210). The pH value reaches the pZc point when the pH does not change when some supplementary reactants are added (Figure 1). Applying this method, the pZc value = 6.7 was calculated, which is the experimental pZc point. This pH value has to be considered as a border in the adsorption/desorption process: absorption below this pH and desorption above this pH limit.

### 2.3. Preliminary Sc(III) Adsorption Studies

To select the best category of the low-temperature-activated alumina products from Table 1, with the ability to perform well in scandium recovery processes, a preliminary experimental study was performed, targeting the adsorption from 10 g Sc(III)/L solutions. Thus, 0.1 g of each material (20 samples from Table 1) was weighed and added over 25 mL of Sc(III) solution of 10 mg/L concentration (using Sc(NO3)3 standard, Merck). The samples were stirred in a JULABO SW 23 thermostatic bath for 60 min at 298 K. The residual Sc(III) concentration was determined with the ICP-MS equipment (Plasma Quant 9100 Analytic Jena), and the adsorption capacity was calculated with the relation:

$$q=\frac{\left(\mathrm{Ci}-\mathrm{Crez}\right)*V}{m}\;[\mathrm{mg}/g]$$

where Ci is initial concentration, mg/L; Crez is residual concentration, mg/L; V is volume of solution, L; m is sample mass, g.

**Figure 2:**
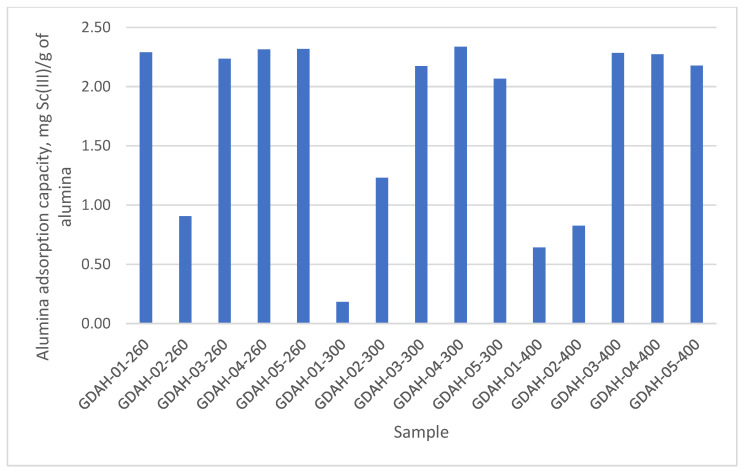
Preliminary scandium adsorption test on low-temperature-activated alumina products.

The experimental data obtained from the preliminary scandium adsorption test on the low-temperature-activated alumina samples are presented in Figure 2. From these data, it can be observed that the 0–10 µm fraction (GDAH-4-300, the smallest size dimension of all GDAH samples) has the highest adsorption capacity for Sc(III). Even if the differences in sample adsorption capacity—when compared to the other samples—are not quite significant. It should be noted that in all experiments, the fractions under 10 and under 20 microns are more reactive than the others. Maybe, for other equilibrium concentrations of scandium, these differences might be more evident. However, this peculiarity is always noted for other proprieties of low-temperature-activated alumina products.

Sample GDAH-04-300 (fraction 0–10 µm), highlighted for the increased thermal stability and good adsorption capacity of the low-temperature-activated alumina products, was selected as a working model for all measurements included in previous papers concerning adsorption [60]. Actually, the absorption capacities of these samples represent the maximum capacities for all the low-temperature-activated alumina products saturated with Sc(III) from a Sc(III) solution with a concentration of 10 mg/L at 298 K. Moreover, the higher adsorbent capacity, assumed for samples GDAH-03-300 and GDAH-04-300, is a direct effect of both mechanical activation (milling and classification) and thermal activation as driving factors in the conversion process of the aluminium hydroxide into low-temperature-activated alumina products [54,58,59,60,64,65].

### 2.4. Effect of the pH on Scandium Absorption Process

**Figure 3:**
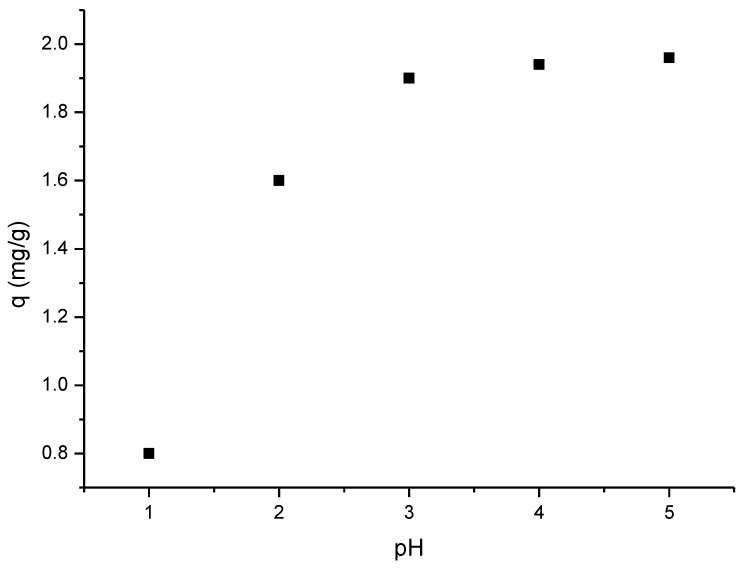
The influence of pH on the adsorption capacity of GDAH-04-300 material.

In order to establish the optimum pH of the Sc(III) adsorption process, the pH was varied in the range of 1 to 5. Studies cannot be conducted at pH > 5 because scandium precipitates as hydroxide. For this study, 0.1 g of each adsorbent sample was prepared for contact with 25 mL solutions of Sc(III) 10 mg/L. Each prepared sample was transferred in a shaking bottle and mixed for 60 min at 298 K in a thermostating bath. The pH of the solutions was set by using HNO_3_ and NaOH solutions, which had concentrations in the range of 0.1–1 N. These solutions were also obtained by the dilution of HNO_3_ 63% (Carl Roth) and dissolution of NaOH pellets (Merck Sigma Aldrich). From the experimental data presented in Figure 3, it was concluded that increasing the pH improved the adsorption capacity of GDAH-04-300. The maximum adsorption capacity was found at pH = 3, i.e., ~2 mg Sc(III)/g adsorbent.

### 2.5. Effect of Contact Time and Temperature on the Scandium Adsorption on Sample GDAH-04-300

**Figure 4:**
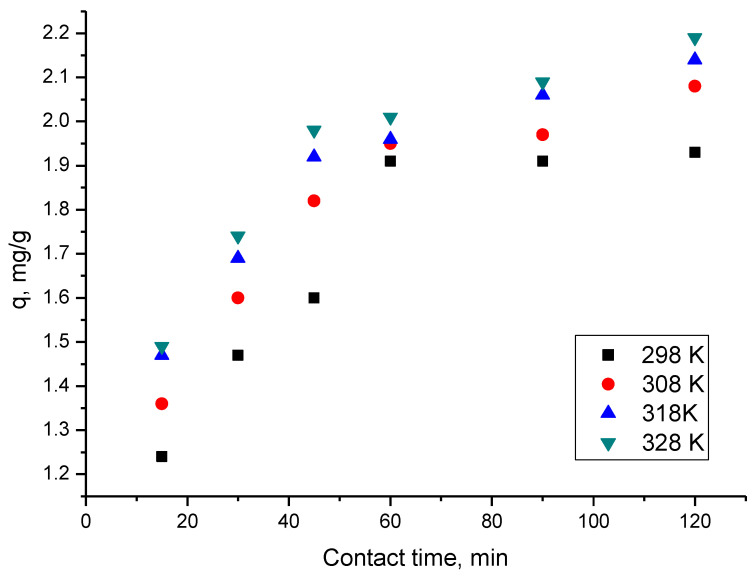
Contact time and temperature effects on the adsorption capacity of scandium on sample GDAH-04-300.

Contact time and temperature are common parameters in all adsorption studies. To perform the experiment, 0.1 g samples of GDAH-04-300 were accurately weighed and mixed with 25 mL of 10 g/L Sc(III) solution. The samples were successively placed in a thermostatic water bath at temperatures of 298 K, 308 K, 318 K and 328 K, time periods varying in the order of 15, 30, 45, 60, 90 and 120 min with stirring at 200 rpm. After reaching any point in the temperature and time program, the samples were quickly filtered. After homogenisation, the samples were analysed quickly, and then the residual concentrations and the amounts of scandium retained on the surface of the GDAH-04-300 product were calculated. The influence of the contact time at four temperature values (298 K, 308 K, 318 K and 328 K) on the adsorption capacity of the GDAH-04-300 material is shown in Figure 4.

From these experimental data, significant increases in adsorption capacity with the duration of contact time can be seen. After 90 min, the adsorption capacity reaches maximum equilibrium values, which are about 2 mg/g of GDAH-04-300. Furthermore, it was observed that as the temperature increases, the adsorption capacity of the GDAH-04-300 material increases. The increase is insignificant, so the adsorption process is recommended to be performed at 298 K.

### 2.6. Kinetic Studies

The efficiency of the adsorption process depends on how the material with adsorbent properties behaves kinetically and thermodynamically. A solid material with a high adsorption capacity, but a low reaction rate, is not a suitable choice because it requires a longer time for the adsorbed molecules to penetrate into the adsorbent particles. On the other hand, an adsorbent with a high reaction rate, but low adsorption capacity, is not beneficial either as it requires a large amount of adsorbent, which would lead to additional costs. An adsorbent with a high adsorption capacity and a high reaction rate is the ideal material for the adsorption process [66].

Kinetic studies provide information about the optimum conditions, the adsorption mechanism and the rate of the adsorption process (mass transfer processes and chemical reactions).

The most commonly used kinetic models to describe the adsorption process are the pseudo-first-order kinetic model (Lagergren model) and the pseudo-second-order kinetic model (Ho and McKay model) [67]. These kinetic models are described by the following equations:

- Pseudo-first-order kinetic equation (Lagergren model) [68]:

$$\ln\left(q_{e}-q_{t}\right)=\ln q_{e}-k_{1}t$$

where q_e_ is the equilibrium adsorption capacity (mg/g); q_t_ is the adsorption capacity at a specific time, t (mg/g); k_1_ is the pseudo-first-order rate constant (1/min); t is contact time (min).

- Pseudo-second-order kinetic equation (Ho and McKay model) [69]:

$$\frac{t}{q_{t}}=\frac{1}{k_{2}q_{e}^{2}}+\frac{1}{q_{e}}$$

where q_e_ is the equilibrium absorption capacity (mg/g); q_t_ is the adsorption capacity at a specific time, t (mg/g); k_2_ is the pseudo-second-order rate constant (g/mg·min); t is contact time (min).

By plotting the linear dependence ln(q_e_ − q_t_) = f(t), a line is obtained, from whose slope the pseudo-first-order rate constant, k_1_, and the adsorption capacity, q_e,calc_ are calculated.

In the case of the pseudo-second-order kinetic model, the linear dependence between t/qt and t is plotted. From the slope of the line obtained, the adsorption capacity, q_e,calc_, and the pseudo-second-order rate constant, k_2_, are calculated.

With the help of the calculated kinetic parameters, it is possible to establish the model that best describes the adsorption process of the adsorbate on the material with adsorbent properties depending on the value of the correlation coefficients, R^2^. The value of the correlation coefficient should be as close as possible to 1 for a certain model to be assigned to the studied adsorption process. This correlation is influenced by pH, temperature and the reactions taking place during the adsorption process [70,71,72].

The adsorption process on porous materials can be described by a mechanism of three consecutive steps: (i) mass transfer of the ions to be adsorbed (the adsorbate) by diffusion from the bulk fluid phase to the adsorbent/solution interface (film diffusion); (ii) mass transfer of the adsorbate from the interface into its pores (intraparticle diffusion); and (iii) adsorption of the adsorbate inside the adsorbent pores by physical or physicochemical adsorption [73,74].

To distinguish whether film diffusion or intraparticle diffusion is the rate-controlling step, the experimental data obtained from kinetic adsorption studies are interpreted using the Weber and Morris model [75]:

$$q_{t}=k_{\mathrm{dif}}\cdot t^{0.5}+C\;$$

where q_t_ is the adsorption capacity at time t, mg/g; k_diff_ is the rate constant for intraparticle diffusion, mg/g·min^1/2^; C is the constant correlated with the thickness of the liquid film surrounding the adsorbent particles.

However, for intraparticle diffusion processes to be the only rate-controlling step, it is necessary that the plot of dependence of q_t_ and t^0.5^ be a linear curve, or with as good of linearity as possible, which passes through the origin (C = 0). Otherwise, both intraparticle diffusion and film diffusion influence the adsorption kinetics. Moreover, a negative value of C indicates that film diffusion influences kinetics.

**Figure 5:**
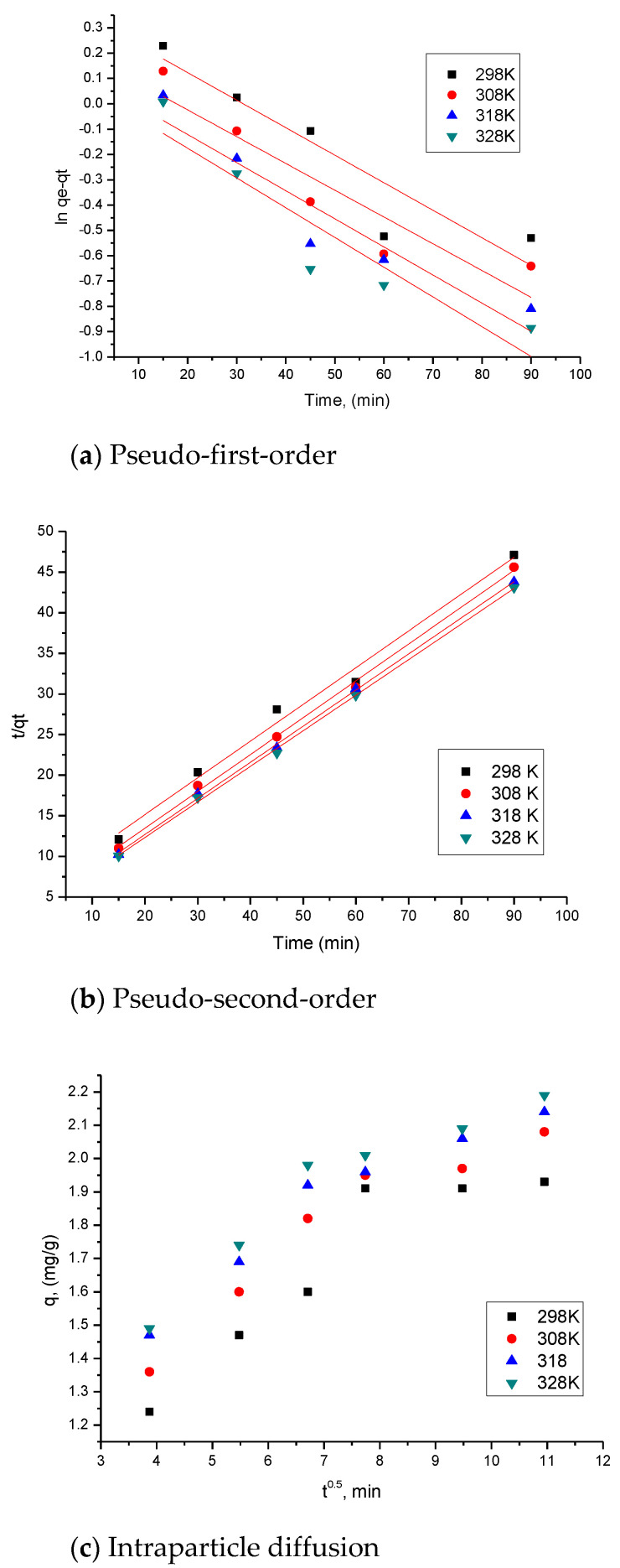
Kinetic studies. The values of rate constants, k, adsorption capacity, as well as for K_diff_ and C parameters from the modelling, are shown in Table 3. Also shown in the same table are the values of the regression coefficient, R^2^.

The experimental data obtained were modelled using the equations of pseudo-first-order and pseudo-second-order kinetic models (Figure 5a,b).

In order to determine whether film diffusion or intraparticle diffusion is the rate-controlling step, kinetic parameters were calculated using the Weber and Morris kinetic model, investigating intraparticle diffusion (Figure 5c).

It can be concluded that the experimental data fit very well with the pseudo-second-order kinetic model. This is sustained by the value of the regression coefficient, R^2^~1. Moreover, q_e,calc_ on the basis of the pseudo-second-order isotherm has close values to q_e,exp_. The temperature influences parameter values, k_2_, q_e,calc_, but not significant enough to consider it required to be treated at temperatures higher than 298 K.

At the same time, it was noticed that the adsorption mechanism of Sc(III) is carried out in several steps because the lines obtained by graphing the linear dependence of q_t_ = f(t^1/2^) at different temperatures do not pass through the origin (C = 0). Therefore, we can say that both intraparticle diffusion and film diffusion influence the kinetics of adsorption.

From the data presented in Table 3, one can observe that as the temperature rises, the K_diff_ value increases. It can also be noticed that the diffusion constants specific to stage 1 are higher than the diffusion constants specific to stage 2, which allows us to state that the speed determinant is stage 1, and the process is slower in stage 2 [74].

**Figure 6:**
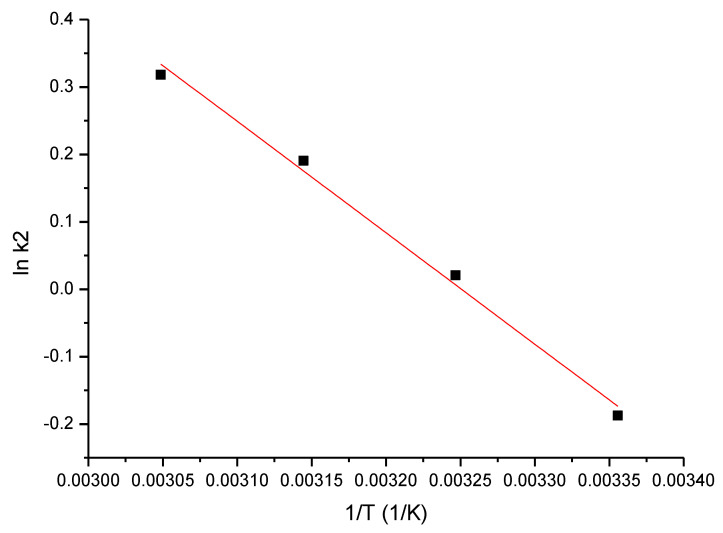
Lnk_2_ vs. 1/T.

For Sc(III) adsorption on GDAH-04-300 material, the activation energy, E_a_, was calculated using the Arrhenius equation and the rate constant, k_2_, using the pseudo-second-order kinetic model (Figure 6).

Further, it can be observed that the activation energy (1.4 kJ/mol) is <40 kJ/mol, which indicates that the adsorption process is physical in nature [76].

### 2.7. Thermodynamic Studies

The evaluation of the activation energy value (E_a_) gives us information about how the adsorption process takes place, which can be physical or chemical [77].

E_a_ is determined by the Arrhenius equation, using the rate constant k_2_ obtained from the kinetic model describing the adsorption process in this study:

$$\ln k_{2}=\ln A-\frac{E_{a}}{\mathrm{RT}}$$

where k_2_ is the rate constant, g/min∙mg; A is Arrhenius constant, g∙min/mg; E_a_ is activation energy, kJ/mol; T is absolute temperature, K; R is the ideal gas constant, 8.314 J/mol∙K. By graphing the linear dependence between ln k_2_ and 1/T, the activation energy of the adsorbent was calculated using the slope of the line obtained. To explain the adsorption mechanism, the Gibbs free energy (ΔG°) value was calculated using the Gibbs–Helmholtz equation [78]:

ΔG°=ΔH°−TΔS°

where ΔG° is the Gibbs free energy variation (kJ/mol); ΔH° is enthalpy standard variation (kJ/mol) ΔS° is entropy standard variation (J/mol·k); T is absolute temperature (K).

Using the van’t Hoff equation, the standard enthalpy and standard entropy values associated with the adsorption process are determined. The two parameters are obtained from the slope of the line, more exactly from the ordinate at the inference of the linear dependence between ln K_d_ și 1/T (as can be seen from the following equation).

$$\ln K_{d}=\frac{\Delta S{}^{\circ}}{R}-\frac{\Delta H{}^{\circ}}{\mathrm{RT}}$$

where K_d_ is the equilibrium constant; ΔS° is the entropy standard variation (J/mol·k); ΔH° is the enthalpy standard variation (kJ/mol); T is absolute temperature (K); R is the ideal gas constant (8.314 J/mol∙K)

The equilibrium constant of the adsorption process is the ratio of the equilibrium adsorption capacity, q_e_, to the equilibrium concentration, C_e_.

$$K_{d}=\frac{q_{e}}{C_{e}}$$

The energy required to bring the adsorbate into contact with the surface of the sorbent is given by the positive value of the standard enthalpy (ΔH°). The affinity manifested by adsorbates toward sorbent is evidenced by the occurrence of electrostatic or complexation interactions in an endothermic process (ΔH° < 50 kJ/mol, physical-sorption) or by the occurrence of chemical bonds in an exothermic process (ΔH° > 50 kJ/mol, chemosorption) [79].

The negative value of the Gibbs free energy variation, ΔG°, obtained from the experimental data indicates that the adsorption process is spontaneous at all temperatures and natural. Otherwise, if the ΔG is positive, nonspontaneous or if ΔG = 0, the process is reversible (at equilibrium). It is relevant to know that the spontaneity of a process may depend upon the temperature of the system. The adsorption speed at the adsorbent/solution interface is indicated by the positive value of the standard entropy variation, ΔS°. All molecules possess a certain amount of energy, which can be in the form of kinetic/potential energy. The activation energy can be interpreted as the minimum amount of kinetic energy that the reactants must have in order for chemical transformations to take place so that adsorption at the liquid/solid interface becomes possible. In order to understand the adsorption mechanism, it is necessary to determine the intermolecular forces that drive the process [79].

**Figure 7:**
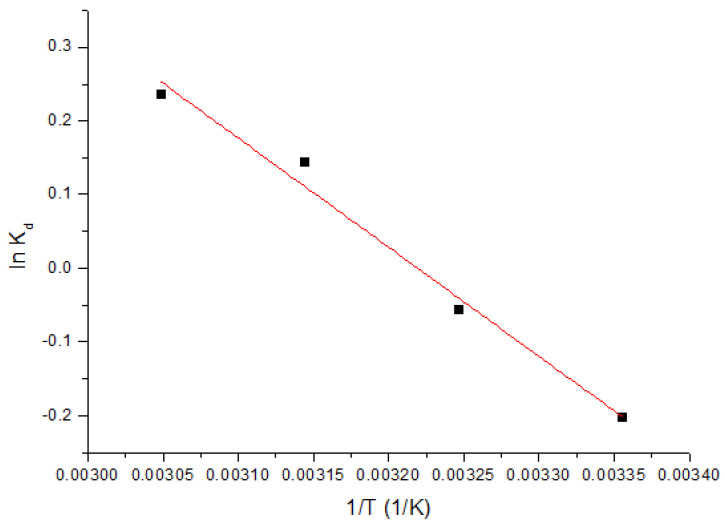
Thermodynamic studies.

Using the van’t Hoff equation and from the equation of the line obtained by plotting the linear dependence ln K_d_ = f(1/T), as shown in Figure 7, standard entropy variation ΔS° and standard enthalpy variation ΔH° can be calculated.

In Table 4, we present the measured thermodynamic parameters at three different temperatures.

From the output data, we can observe that ΔH° has a positive value, which means that the adsorption process is endothermic. We can also notice that ΔG° has negative values and grows in absolute value with temperature increase, which indicates that the adsorption process is spontaneous and influenced by temperature.

The fact that the ΔS° value was positive indicates that the adsorption process is enhanced, taking place at the interface of the GDAH-04-300/solution material with Sc(III) content.

### 2.8. Equilibrium Adsorption

The adsorption capacity at equilibrium is an important parameter for proper analysis and design of the adsorbent–adsorbate system [80]. The adsorption isotherm defines the relationship between adsorbent adsorption capacity and adsorbate concentration at equilibrium at a constant temperature, which explains the interactions between adsorbate and adsorbent. This way, the adsorption isotherm models can provide information about the mechanism of the adsorption process and also about the maximum adsorption capacity of the adsorbent material.

Lately, linear regression analysis has been one of the most applied analytical techniques to define the best adsorption models because it measures the adsorbate distribution, analyses the adsorption system and tests the consistency of the theoretical assumptions of the adsorption isotherm model. The isotherm models used in the present study are: one-parameter isotherms (Henry), two-parameter isotherms (Langmuir, Freudlich, Dubinin-Radushkevich, Temkin, Flory-Huggins, Halsey, Hill, Jovanovich, Elovich, Kiselev, Hill-Deboer, Fowler-Guggenheim, Harkin-Jura), three-parameter isotherms (Redlich-Peterson, Toth, Sips, Khan, Koble-Carrigan, Langmuir-Freundlich, Radke-Prausniiz, Jossens), four-parameter isotherms (Fritz-Schlunder, Baudu, Weber-Van Vliet, Marczewski-Jaroniec) and five-parameter isotherms (Fritz-Schlunder) [81]. The determination of the Langmuir isotherm was based on the argument that: (i) the active centres on the surface of the solid adsorbent are constant in number, identical and uniformly distributed on the surface; (ii) each active centre can adsorb only one molecule so that the adsorption layer must be precisely monomolecular and the adsorption aims at a limit matching the occupancy of all the active centres on the surface; (iii) the adsorption temperature of the active centres is assumed to be equal and independent of the degree of surface coverage, and no interactions occur between the neighbouring molecules [82].

The nonlinear form of the Langmuir equation is [83]:

$$q_{e}=\frac{q_{L}K_{L}C_{e}}{1+K_{L}C_{e}}$$

where q_e_ is the maximum adsorption capacity (mg/g); C_e_ is the equilibrium concentration of a metallic ion in solution (mg/L); q_L_ is the Langmuir maximum adsorption capacity (mg/g); K_L_ is the Langmuir constant. R_L_ is the characteristics of the Langmuir isotherm and can be expressed by a dimensional constant called the separation factor. R_L_, also known as the equilibrium parameter, is calculated using the equation:

$$R_{L}=\frac{1}{1+K_{L}C_{o}}$$

where R_L_ is the separation factor; K_L_ is the Langmuir constant (L/mg); C_o_ is the initial concentration of adsorbate (mg/L).

The Freundlich isotherm is relevant to the adsorption process, which takes place on a heterogeneous surface. The equation defines the heterogeneous surface of the adsorbent and the energy and exponential distribution of the active centres [84].

The nonlinear form of the equation is [85]:

$$q_{e}=K_{F}C_{e}^{1/n_{F}}$$

where q_e_ is the maximum adsorption capacity (mg/g); C_e_ is the equilibrium concentration of a metallic ion in solution (mg/g); K_F_ și n_F_ are the characteristic constants that can be associated with the relative adsorption capacity of the adsorbent and the intensity of the adsorption process.

The value of n indicates the degree of non-linearity between the solution concentration and the adsorption process, thus:

- If *n* = 1, then adsorption is linear;
- If *n* < 1, then adsorption is a chemical process;
- If *n* > 1, then adsorption is a physical process.

Furthermore, it has been established that for n between 1 and 10, the adsorption process is very good [86,87].

The Sips isotherm is a combination of the Langmuir and Freundlich isotherms, which is expressed by the nonlinear form of the equation [88]:

$$q_{e}=\frac{q_{S}K_{S}C_{e}^{1/n_{S}}}{1+K_{S}C_{e}^{1/n_{S}}}$$

where q_S_ is the maximum adsorption capacity (mg/g); C_e_ is the concentration of adsorbate at equilibrium (mg/L); K_S_ is a constant related to the adsorption capacity of the adsorbent; n_S_ is a heterogeneity factor.

In the case of low adsorbate concentrations, the adsorption process can be assumed to be modelled by the Freundlich isotherm, and at higher adsorbate concentrations, it can be modelled by the Langmuir isotherm [84].

Using the parameters of the Sips isotherm, a separation factor, R_S_, which is a dimensionless equilibrium parameter, is calculated using the equation:

$$R_{S}=\frac{1}{1+K_{S}C_{o}^{1/n_{S}}}$$

where: R_S_—separation factor; K_S_—Sips constant; n_S_—heterogeneity factor; C_o_—initial concentration (mg/L).

The value of *R_S_* allows the assessment of the type of adsorption and is an important characteristic of the Sips isotherm.

If R_S_ >1, the adsorption process is unfavourable, the isotherm has a concave shape; if R_S_ = 1, the isotherm is linear; if 0 < R_S_ < 1, the isotherm has a convex shape and the adsorption process is favourable; and if R_S_ = 0, the adsorption is irreversible.

The isotherms are represented as linearised equations q_e_ = f(C_e_), and the specific parameters of each isotherm, used to model the experimental data, are calculated from the slopes and based on the intersection of the lines (the origin).

**Figure 8:**
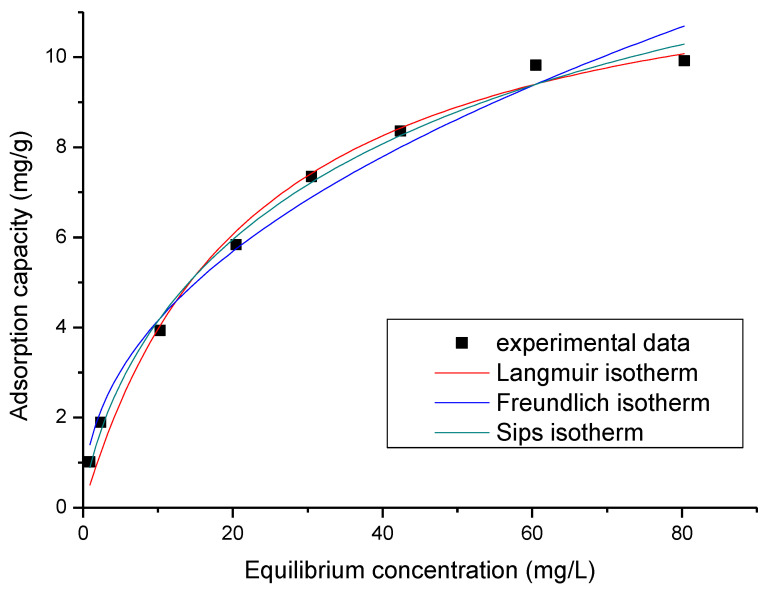
Equilibrium studies.

In Figure 8, the modelled data are shown.

From these data, it can be observed that as the initial Sc(III) concentration increases, the adsorption capacity of the GDAH-04-300 material also increases, reaching equilibrium at 80 mg/L Sc(III), when the maximum adsorption capacity is ~9.4 mg Sc(III)/g material. From a previous study, this value is comparable to that obtained in the case of silver adsorption (10.2 mg Ag(I)/g), which suggests that the GDAH-04-300 material behaves in a similar way [60]. The specific parameters of each isotherm used to model the experimental data were calculated from the straight-line slopes using the y-intercept ordinate (Table 5).

The correlation between the equilibrium concentration (C_e_) of Sc(III) and the adsorption capacity indicates that as the equilibrium concentration increases, the adsorption capacity also increases until equilibrium is achieved, setting the maximum adsorption capacity experimentally determined, q_e,exp_ (~9.8 mg Sc(III)/g).

According to the data in Table 3, it is concluded that the most accurate model to describe the adsorption process is the Sips isotherm since the regression coefficient, R^2^, is closest to 1 (R^2^ = 0.9915) and the theoretical adsorption capacity is ~10.1 mg Sc(III)/g.

## 3. Materials and Methods

### 3.1. Samples Materials

The samples of aluminium hydroxide were collected from the last test of the new production line at Alum SA Tulcea, Romania, which was built up by the implementation of the project “Endow the Research and Development Department of SC ALUM SA Tulcea with independent and efficient research facilities to support the economic competitiveness and business development”, a project co-funded by the European Regional Development Fund through the Competitiveness Operational Program 2014–2020. This line of production can deliver new grades of aluminium hydroxide dried, milled and classified with variable particle size distribution. The representative samples of the above precursor were carefully selected for adequate thermal treatments in order to convert the precursor into low-temperature-activated alumina products with significant adsorption properties. The particle size and temperatures of calcination samples are presented in Table 6. The temperature and thermal process have been chosen with a defined purpose to promote some specific properties of adsorbents: low-temperature transitional phases, amorphous phases with a large specific surface, etc. Actually, the samples were first dried at 60 °C for 24 h, then heated in an electric furnace (in air atmosphere) at 260 °C, 300 °C and 400 °C, with a heating rate of 5 °C/min, and subsequently held for thermal stabilisation for 2 h at the above-mentioned temperature values. Afterwards, the samples were slowly cooled in the oven until reaching room temperature.

Concerning the particle size and thermal treatment, physical and technical properties of the samples from Table 6, it should be noted that all the analysed proprieties of these materials are controlled by two driving factors: particle size distribution and calcination temperature. The dependence is so evident that the data for samples GDAH-03 (under 20 µm) and GDAH-04 (under 10 µm) are completely different from the same parameters for samples GDAH-01 and GDAH-02. The particular properties of these low-temperature-activated alumina products have been studied in our previous papers [54,58,59,60,65]. Therefore, all the scandium adsorption data can be analysed on the basis of rigorous knowledge about the sample mineralogy and chemistry, as well as about all other sample properties.

### 3.2. Scandium Adsorption Capacity of the Low-Temperature-Activated Alumina Products

In this study, the scandium adsorption capacity was the most important parameter of low-temperature-activated alumina products as adsorbents. The method was applied in several ways depending on the purpose of its measurement. The maximum values are used to compare the different adsorption performances, but under restricted circumstances (as multiple ionic concentrations and the solutions pH, multiple component liquid phases, etc.), some partial values are also admitted. Besides, in this study, there was admitted, as a term of comparison, the adsorbent capacity values of different low-temperature-activated alumina products determined using 10 mg/L scandium nitrate solution for the selection of the best adsorbent from all the low-temperature-activated alumina products. In other instances, the measurements consist of mixing for 60 min at 298 K, appropriate quantities of any of the alumina product samples from Table 6 in 25 mL Sc(III) solution of 10 mg/L concentration (standard Sc(NO_3_)_3_, Merck). Finally, the residual concentration of Sc(III) was measured by the ICP-MS (PlasmaQuant 9100 Analytic Jena – Analytik Jena GmBH, Jena, Germany) method and the computations were made with the formula given in our previous paper [60]. This method has been used in several variants throughout this paper.

### 3.3. Analysis Equipment

Data procurement in all categories of measurements required by this work is similar to the one used in our previous papers, including the analysis equipment [54,58,59,60].

## 4. Conclusions

From the experimental data presented in this study, it was concluded that the 0–10 µm particle size (the smallest) GDAH samples possess good adsorption capacity for scandium. This was an expected result because the same GDAH sample exhibited particularly improved physical and technical properties due to its small size dimension and crystalline/amorphous ratio, as was shown in our previous papers. Its special properties are related to the effects of three dynamic factors acting during the low-temperature activation: temperature (300 °C), rate of heating and advanced grinding. In order to achieve the highest adsorption capacity for the recovery of Sc(III), adsorption studies were carried out following the influence of specific parameters such as S:L ratio, pH, contact time, temperature and initial Sc(III) concentration. Therefore, the working conditions were: pH = 3, contact time = 45 min, temperature = 298 K, initial concentration = 80 mg Sc(III)/L. In these conditions, the maximum adsorption capacity was ~9.8 mg Sc(III)/g material.

The adsorption capacity of the material obtained by experimental measurements and confirmed by modelling (~9.8 mg Sc(III)/g) is comparable to the adsorption capacity of other metal ions, e.g., Ag I (~10.2 mg/g).

Scandium recovery by adsorption onto GDAH-04-300 material is a spontaneous, endothermic physical process occurring at the interface of GDAH-04-300/solution containing scandium and whose kinetics are influenced by both interpacticle diffusion and film diffusion.

## Figures and Tables

**Table 1:** Specific surface area and pore width measurements for the initial samples and after thermal treatment at 260 °C, 300 °C and 400 °C for 2 h.

Samples	BET Specific Area (m^2^/g)	Langmuir Specific Area (m^2^/g)	Average Pore Width (nm)
**GDAH-01**	3.5419	5.7029	4.68861
**GDAH-01_260 °C**	2.8412	4.2435	4.67366
**GDAH-01-300 °C**	41.9800	61.0822	2.70180
**GDAH-01-400 °C**	3.1220	270.002	3.15536
**GDAH-02**	5.9596	8.9199	10.9876
**GDAH-02-260 °C**	10.6208	15.5593	5.7378
**GDAH-02-300 °C**	36.3853	52.7869	3.7164
**GDAH-02-400 °C**	234.4518	345.6202	3.2249
**GDAH-03**	10.3375	16.0231	7.1186
**GDAH-03_260 °C**	10.3094	15.5083	5.9023
**GDAH-03_300 °C**	65.5179	95.2101	3.1337
**GDAH-03_400 °C**	241.9623	356.6276	3.3867
**GDAH-04**	9.4725	14.0771	7.4804
**GDAH-04_260 °C**	19.4569	28.2606	5.4720
**GDAH-04_300 °C**	6.9195	10.0146	3.7566
**GDAH-04_400 °C**	238.6443	350.5961	3.2303
**GDAH-05**	2.2240	7.3240	4.3304
**GDAH-05_260 °C**	2.2964	3.7623	6.1816
**GDAH-05_300 °C**	20.8556	30.3694	3.8894
**GDAH-05_400 °C**	181.5672	267.8954	3.5443

**Table 2:** Effect of the calcination temperature on the phase composition and crystallinity.

Mineral Name	Gibbsite (%)	Boehmite (%)	Gamma-Al_2_O_3_ (%)	Crystallinity (%)
Formula	Al(OH)_3_	γ-AlOOH	γ-Al_2_O_3_	
**GDAH-01**	41.65	0	0	58.35
**GDAH-02**	62.54	0	0	37.46
**GDAH-03**	61.67	0	0	38.33
**GDAH-04**	62.59	0	0	37.41
**GDAH-02**	90	0	1.6	62.54
**GDAH-02-260**	91.8	8	0.1	50.57
**GDAH-02-300**	78.8	13.1	1.5	41
**GDAH-02-400**	15.7	35.7	48.3	21.4
**GDAH-03**	97.2	0	0	61.67
**GDAH-03** **-260**	93.2	6.2	0.5	48.21
**GDAH-03** **-300**	80	11	1.3	43.75
**GDAH-03** **-400**	0.2	26.3	57.7	18.30
**GDAH-04**	88.9	0	0.8	62.59
**GDAH-04** **-260**	89.3	4.9	1.4	55.85
**GDAH-04** **-300**	76.7	10.4	2.5	49.38
**GDAH-04** **-400**	8.7	30.4	57.1	22.12
**GDAH-05**	99.9	0	0	55.56
**GDAH-05** **-260**	84.1	15.9	0	51.76
**GDAH-05** **-300**	73.8	26.2	0	45.75
**GDAH-05** **-400**	0.2	48.8	50.9	26.68

**Table 3:** Kinetic parameters for the adsorption of Sc(III) onto GDAH-04-300.

Pseudo-First-Order				
Temperature (K)	*q*_e,exp_ (mg g^−1^)	*k*_1_ (min^−1^)	*q*_e,calc_ (mg g^−1^)	R^2^
298	1.91	0.0077	1.00	0.7889
308	1.97	0.0089	1.04	0.8941
318	2.06	0.0246	1.13	0.921
328	2.09	0.0304	1.24	0.9127
Pseudo-Second-Order				
Temperature (K)	*q*_e,exp_ (mg g^−1^)	*k*_2_ (g mg^−1^∙min^−1^)	*q*_e,calc_ (mg g^−1^)	R^2^
298	1.91	0.83	2.14	0.9950
308	1.97	1.02	2.25	0.9988
318	2.06	1.21	2.29	0.9993
328	2.09	1.37	2.33	0.9991
Intraparticle Diffusion Model (IPD)				
Temperature (K)	K_diff_ (mg·g^−1^ min^−1/2^)	C		R^2^
298	2.51	12.52		0.8797
308	3.92	12.89		0.8860
318	4.04	13.02		0.8835
328	4.36	13.56		0.7848

**Table 4:** Thermodynamic parameters for adsorption of Sc(III) onto GDAH-04-300.

ΔH° (kJ/mol)	ΔS° (J/mol∙K)	ΔG° (kJ/mol)				R^2^
12.32	39.66	298 K	308 K	318 K	328 K	0.9856
		−11.80	−12.20	−12.60	−12.99	

**Table 5:** Parameters of isotherm model for adsorption Sc(III) onto GDAH-04-300.

Langmuir isotherm			
q_m,exp_ (mg/g)	K_L_ (L/mg)	q_L_ (mg/g)	R^2^
9.82	0.044	12.91	0.9856
Freundlich isotherm			
K_F_ (mg/g)	1/n_F_		R^2^
1.46	0.45		0.9802
Sips isotherm			
K_S_	q_S_ (mg/g)	1/n_S_	R^2^
0.26	10.1	0.06	0.9915

**Table 6:** Low-temperature aluminium hydroxide sample grades.

Sample	GDAH-01	GDAH-02	GDAH-03	GDAH-04	GDAH-05
Particle size	<45 µm = 5.7% >150 mm 6.4%	<45 µm 98.29%	<20 µm 92.13%	<10 µm 76.28%	<45 µm = 5.0% >150 = 3.42%
LOI	34.62	34.62	34.61	34.62	34.58
Moisture	0.082	0158	0.134	0180	0.081
Temperature 1	GDAH-0	GDAH-01	GDAH-01	GDAH-01	GDAH-01
Temperature 2	GDAH-01 260	GDAH-01 260	GDAH-01 260	GDAH-01 260	GDAH-01 260
Temperature 4	GDAH-01 300	GDAH-01 300	GDAH-01 300	GDAH-01 300	GDAH-01 300
Temperature 5	GDAH-01 400	GDAH-01 400	GDAH-01 400	GDAH-01 400	GDAH-01 400
